# Correlation between sialyl Tn antigen and lymphatic metastasis in patients with Borrmann type IV gastric carcinoma.

**DOI:** 10.1038/bjc.1995.39

**Published:** 1995-01

**Authors:** Y. Kakeji, Y. Maehara, M. Morita, A. Matsukuma, M. Furusawa, I. Takahashi, T. Kusumoto, S. Ohno, K. Sugimachi

**Affiliations:** Department of Gastroenterologic Surgery, National Kyushu Cancer Center, Fukuoka, Japan.

## Abstract

**Images:**


© 1995 Stockton Press All rights reserved 0007-0920/95 $9.00

### Correlation between sialyl Tn antigen and lymphatic metastasis in patients with Borrmann type IV gastric carcinoma

Y Kakeji[1], Y Maehara[2], M Morita[1], A Matsukuma[1], M Furusawa[1], I Takahashi[2], T Kusumoto[2], S Ohno[2] and K Sugimachi[2]

[1]*Department of Gastroenterologic Surgery, National Kyushu Cancer Center, and* [2]*Department of Surgery II, Faculty of Medicine, Kyushu University, Fukuoka, Japan.*

**Summary** The expression of sialyl Tn (STn) antigen in 180 patients with Borrmann type IV gastric carcinomas was examined immunohistochemically. The rate of positive STn staining was 32% (57/180) for the primary tumours, and this positive staining correlated well with tumour extension, lymph node metastasis ($P<0.05$) and peritoneal dissemination ($P<0.01$). One-third (5/15) of patients with positive STn-staining cancer cells had a high level of serum STn. Lesions with positive STn staining were related to a lower survival rate for the patients ($P<0.05$). Proliferative activity of the tumour, as measured by proliferating nuclear antigen (PCNA) labelling percentage and argyrophilic nucleolar organiser region (AgNOR) count, was significantly higher (41.5 ± 13.0%, 3.78 ± 0.98) in the STn-positive group than in the STn-negative group (34.2 ± 13.2%, 3.48 ± 0.85) ($P<0.01$, $P<0.05$ respectively). Estimating STn antigen may be useful for predicting the likelihood of lymph node metastasis or peritoneal dissemination and the clinical prognosis for patients with Borrmann type IV gastric carcinoma.

**Keywords:** sialyl Tn antigen; Borrmann type IV; gastric carcinoma; lymphatic metastasis

Despite advances in diagnostic and surgical management, patients with Borrmann type IV gastric carcinoma have a poor prognosis. Advanced carcinoma of the stomach can be classified, based on Borrmann's criteria, into one of four types (Borrmann, 1926). Borrmann type I carcinoma is a polypoid fungating, type II ulcerative lesion with elevated and distinct borders, and type III is ulcerative but with indistinct borders. Borrmann type IV carcinoma is a diffuse malignant lesion with indistinct borders, and is usually identified only at a very advanced stage (Borchard, 1990; Maehara *et al.*, 1992). The lack of sharp borders of the tumour can make for an underestimation of size. The clinical course is usually unfavourable and the 5 year survival rates are 0-20% (Furukawa *et al.*, 1988; Maehara *et al.*, 1992). A detailed study focusing on the biological behaviour of these highly malignant carcinomas may perhaps improve the prognosis.

Associated with neoplastic transformation, incomplete synthesis of glycolipids or glycoproteins in cell membranes often occurs, resulting in a storage of precursor structures that, in normal cells, would remain cryptic because of further elongation (Hakomori and Kannagi, 1983; Springer, 1984). Sialyl Tn antigen (STn) is one of these abnormal *O*-linked glycoproteins. The Tn antigen is a precursor of the Thomsen-Freidenreich antigen, the T antigen, and STn is a sialic acid-bound Tn antigen (Hakomori and Kannagi, 1983; Springer, 1984; Kjeldsen *et al.*, 1988). The relationship between STn expression and biological behaviour of cancer cells has been investigated in some human malignancies. In colonic tissues, Itzkowitz *et al.* (1990) reported a poor outcome for STn antigen-positive patients. Kobayashi *et al.* (1992) concluded that a positive STn antigen in sera was an independent predictor of a poor prognosis in patients with ovarian cancer. We reported that elevated serum STn levels correlate with advanced tumour stage and a worse prognosis of patients with gastric cancer (Takahashi *et al.*, 1993, 1994). Immunohistochemically, Ma *et al.* (1993) and Werther *et al.* (1994) reported that expression of STn antigen may be a useful prognostic marker in patients with gastric cancer. However, the question remained as to whether the lower survival rate of patients with higher STn expression reflects a higher tumour burden.

To better understand the biological behaviour of STn-positive cancer cells in the most advanced stage, we performed immunostaining for STn antigen in Borrmann type IV gastric cancer and the relationship between STn expression and clinicopathological features was examined with regard to clinical prognosis. We also analysed cell proliferative activity determined by proliferating nuclear antigen (PCNA) labelling percentage and argyrophilic nucleolar organiser region (AgNOR) count, both serving as a parameter of proliferating cells (Egan & Crocker, 1993; McCormick & Hall, 1992).

### Materials and methods

### *Patients*

The 180 Japanese patients with primary Borrmann type IV gastric cancer studied herein had undergone gastrectomy in the National Kyushu Cancer Center, Fukuoka, Japan, from 1972 to 1990. A thorough histological examination was made on haematoxylin and eosin-stained preparations, and the histological classification was according to the tumour-node-metastasis classification system of the International Union Against Cancer (UICC, 1987). No patient had been given cytotoxic drugs preoperatively. Post-operative adjuvant chemotherapy was prescribed for 171 patients.

### *Immunohistochemical staining for STn*

Sections from paraffin blocks were dewaxed and stained using the avidin-biotin-peroxidase complex method. The primary antibody TKH2, a monoclonal mouse antibody for sialosyl Tn antigen, was kindly provided by Otsuka Assay Laboratory (Tokushima, Japan). The slides were incubated with fresh 0.3% hydrogen peroxide in methanol for 10 min, then washed three times with phosphate-buffered saline (PBS; pH 7.4). Five per cent normal goat serum in PBS was then applied for 15 min. The sections were incubated overnight with TKH2 (dilution 1:50) at room temperature, with biotinylated goat anti-mouse IgG (1:200 for 30 min; Vector Laboratories) and with the avidin-biotin-peroxidase complex (for 30 min; Vector Laboratories). Peroxidase labelling was developed with 3,3'-diaminobenzidine and hydrogen per-

Correspondence: Y Maehara, Department of Surgery II, Faculty of Medicine, Kyushu University, Maidashi 3-1-1, Higashi-ku, Fukuoka 812, Japan
Received 14 March 1994; revised 1 September 1994; accepted 5 September 1994

oxide and the sections were counterstained with haematoxylin.

To ensure the consistency of STn staining between batches, a known positive control gastric carcinoma was included in each round. Negative controls were prepared by substituting normal mouse serum for primary antibody, the results being no detectable staining.

Cellular localisation of the antigenic sites was determined by two investigators without knowledge of clinicopathological information. A double-headed light microscope was used. Scoring was made by examining all low-power optical fields (10 × objective) containing tumour cells and the percentage of antigen-positive cells was estimated. A positive value was recorded if more than 5% of the tumour cells expressed STn antigen.

### *Serum STn levels*

Serum STn levels were measured for 27 patients surgically treated in the period from 1981 to 1986, using a one-step radioimmunoassay kit (S-Tn Otsuka; Otsuka Assay Laboratories, Tokushima, Japan) (Imamura *et al.*, 1989). Competitive binding to the radiolabelled monoclonal antibody TKH2 between serum STn and STn-coated beads was used (an immunoradiometric competitive inhibition assay) (Kjeldsen *et al.*, 1988). Venous blood samples were obtained from patients after an overnight fast and were immediately centrifuged and the serum placed in liquid nitrogen until assay. The cut-off between normal and elevated STn titres was set at $45\ U\ ml^{-1}$; this is the mean + two standard deviations (s.d.) of the STn value in normal volunteers, as reported by Imamura *et al.* (1989).

### *Proliferative activities*

The avidin–biotin–peroxidase complex method was used for PCNA staining, as described elsewhere (Kakeji *et al.*, 1994). The primary antibody was PC10 (Dako, Carpinteria, CA, USA). The PCNA labelling percentage was determined by observing 1000 nuclei in areas of the section with the highest labelling percentage, and the percentage of PCNA-labelled nuclei was used for analysis. For AgNOR staining, the one-step silver colloid method was used. The NOR staining solution was prepared according to the description of Ploton *et al.* (1982). On the AgNOR-stained slides, careful focusing made visible the AgNOR in the nucleus, in the form of black dots. One hundred cells from each lesion were analysed and a mean score of AgNOR count was recorded.

### *Statistical analysis*

Clinicopathological data were stored in an IBM (Armonk, NY, USA) 4381 mainframe computer. The Biomedical Computer Program (BMDP) was used for all statistical analyses (Dixon, 1988). The BMDP P4F and P3S programs were used for the chi-square test, and the Mann–Whitney test was used to compare characteristics between positive and negative groups with STn staining. The BMDP P1L program was used for analysing survival rates, together with the Kaplan–Meier method, and for testing equality of survival curves, using the method of Mantel and Cox. In the statistical analysis, deaths due to causes other than gastric carcinoma were considered censored cases.

### Results

### *STn staining and clinicopathological characteristics*

In the normal gastric mucosa, positive staining for STn was recognised in parietal cells with STn expression in intracellular canalicular membranes. In cancer tissues, the intensity and incidence of staining varied widely from case to case and from area to area within one case. In general, however, the staining was diffusely cytoplasmic, with strong staining associated with the luminal surface (Figure 1). Positive STn staining was evident in 57 (32%) of 180 primary tumours. No obvious relation was found between STn staining and the gender or age of the patient (Table I). All seven patients in whom tumour invasion was confined to the subserosa (T2) showed negative STn staining, and the rate of positive staining increased in proportion to invasion into the deeper layers ($P<0.05$). STn-positive tumours were associated with a higher incidence of metastasis to lymph nodes and peritoneal dissemination than were STn-negative tumours ($P<0.05$, $P<0.01$, respectively).

### *STn staining and serum STn level*

Table II shows the compatibility of histopathological STn staining and serum STn level. Although the patient numbers were too few for a statistical significance, the data do provide interesting information. All 12 with STn-negative cancer cells in tissues had a low level of serum STn, and one-third (5/15) of the patients with STn-positive cancer cells had a high level of serum STn. Table III shows clinicopathological features of patients with immunohistochemically STn-positive cancer cells, according to serum STn levels. In this analysis of 15 cases, there were no obvious features in patients with high STn levels in tissues or in serum. Though not statistically significant, the mean survival time for patients with high serum STn levels ($>45\ U\ ml^{-1}$) was 298 (131–474) days, that is much shorter than the 778 (148–2932) days for those with low serum STn levels ($<45\ U\ ml^{-1}$).

### *STn staining and prognosis*

The overall survival curve is shown in Figure 2a, according to positive or negative STn staining. Those with positive STn cancers showed worse survival rates than did those with negative STn cancers ($P<0.05$). Even in 'curative' cases, there was a tendency toward a shorter survival for those with positive STn cancers (Figure 2b). Table IV shows the pattern of recurrence, based on STn staining. Though the recurrence rate tended to be higher in patients with STn-positive tumours (13/18; 72%) than that in those with STn-negative tumours (38/65; 58%), no obvious difference was recognised in the pattern of recurrences.

### *STn staining and proliferative activity*

The proliferative activity expressed by mean PCNA labelling percentage was 41.5% for STn-positive cases, a value significantly higher than 34.2% for the STn-negative cases ($P<0.01$, Table V). The mean AgNOR count for STn-positive cases (3.77) was also significantly higher than that for STn-negative cases (3.48, $P<0.05$).

**Figure 1** Gastric carcinoma of moderately differentiated type stained with TKH-2 antibody and showing both cytoplasmic and apical reactivity (× 200).

**Table I** Clinicopathological characteristics and STn staining

| *Histological findings* | *STn staining* Negative (n = 123) | Positive (n = 57) | P-value |
|---|---|---|---|
| Sex | | | |
| Men | 70 | 28 | NS |
| Women | 53 | 29 | |
| Mean age (years) | 55.8±11.7 | 58.6±14.0 | NS |
| Tumour size (cm) | 12.2±4.0 | 13.2±4.0 | NS |
| Histological type | | | |
| Well differentiated | 1 | 2 | NS |
| Moderately differentiated | 11 | 9 | |
| Poorly differentiated | 51 | 13 | |
| Signet | 59 | 26 | |
| Mucinous | 1 | 7 | |
| Tumour extension | | | |
| pT2 | 7 | 0 | $P<0.05$ |
| pT3 | 84 | 31 | |
| pT4 | 32 | 26 | |
| Invasion into lymphatics | | | |
| No invasion | 8 | 1 | NS |
| Slight invasion | 49 | 21 | |
| Moderate invasion | 40 | 14 | |
| Severe invasion | 26 | 21 | |
| Venous invasion | | | |
| No invasion | 43 | 15 | NS |
| Slight invasion | 73 | 36 | |
| Moderate invasion | 6 | 5 | |
| Severe invasion | 1 | 1 | |
| Lymph node involvement | | | |
| pN0 | 17 | 4 | $P<0.05$ |
| pN1 | 28 | 8 | |
| pN2 | 59 | 26 | |
| pM1 | 19 | 19 | |
| Peritoneal dissemination | | | |
| Negative | 98 | 31 | $P<0.01$ |
| Positive | 25 | 26 | |
| Metastasis to the liver | | | |
| Negative | 119 | 56 | NS |
| Positive | 4 | 1 | |
| Stage | | | |
| IA | 0 | 0 | $P<0.01$ |
| IB | 1 | 0 | |
| II | 13 | 0 | |
| IIIA | 20 | 4 | |
| IIIB | 32 | 10 | |
| IV | 57 | 43 | |
| Curability | | | |
| Curative | 66 | 17 | $P<0.01$ |
| Non-curative | 57 | 40 | |

**Table II** Compatibility of histopathological STn staining and serum STn level

| *Serum STn level* (U ml$^{-1}$) | *STn staining* Negative | Positive |
|---|---|---|
| <45 | 12 | 10 |
| >45 | 0 | 5 |

**Table III** Clinicopathological characteristics of patients with immunohistochemically STn-positive cancer cells according to serum STn level

| *Histological findings* | *Serum STn level (U ml$^{-1}$)* <45 (n = 10) | >45 (n = 5) |
|---|---|---|
| Sex | | |
| Men | 5 | 4 |
| Women | 5 | 1 |
| Mean age (years) | 50.1±11.1 | 72.8±7.6* |
| Tumour size (cm) | 12.9±3.8 | 14.3±2.4 |
| Histological type | | |
| Well differentiated | 1 | 0 |
| Moderately differentiated | 3 | 1 |
| Poorly differentiated | 2 | 0 |
| Signet | 4 | 2 |
| Mucinous | 0 | 2 |
| Tumour extension | | |
| pT2 | 0 | 0 |
| pT3 | 5 | 3 |
| pT4 | 5 | 2 |
| Invasion into lymphatics | | |
| No invasion | 0 | 0 |
| Slight invasion | 4 | 1 |
| Moderate invasion | 3 | 1 |
| Severe invasion | 3 | 3 |
| Venous invasion | | |
| No invasion | 3 | 1 |
| Slight invasion | 7 | 4 |
| Moderate invasion | 0 | 0 |
| Severe invasion | 0 | 0 |
| Lymph node involvement | | |
| pN0 | 1 | 0 |
| pN1 | 1 | 0 |
| pN2 | 4 | 2 |
| pM1 | 4 | 3 |
| Peritoneal dissemination | | |
| Negative | 5 | 4 |
| Positive | 5 | 1 |
| Metastasis to the liver | | |
| Negative | 9 | 4 |
| Positive | 1 | 1 |
| Stage | | |
| IIIB | 1 | 1 |
| IV | 9 | 4 |
| Curability | | |
| Curative | 3 | 2 |
| Non-curative | 7 | 3 |
| Mean survival (days) | 778 (148-2932) | 298 (131-474) |

*$P<0.01$.

### Discussion

Quantitative differences in cell-surface sialic acid (total cell-surface sialic acid levels) and qualitative changes in sialylated oligosaccharides (presence or absence of a particular oligosaccharide structure or individual glycoconjugates) are commonly associated with metastasis (Yogeeswaran and Salk, 1981; Altevogt *et al*., 1983; Passaniti and Hart, 1988). The finding that STn-positive cancers in primary lesions correlated with lymph node metastasis and with peritoneal metastasis suggests that the STn antigen may be drained specifically by the lymphatic route. The specific drainage route for tumour cells with special antigen was discussed by Tabuchi *et al*. (1990); they stated that CA 19-9, a cancer-associated antigen with sialylated carbohydrates, may be drained primarily by the thoracic duct of the lymphatic system via node metastases or invasive lymphatics. Furthermore, tumours with positive STn staining had a higher proliferative activity than did those with which stained negatively. We have previously reported that tumours with a high proliferative activity often metastasise to lymph nodes (Kakeji *et al*., 1991, 1994). Therefore, this high proliferative activity will probably accelerate the lymphatic spread of cancer cells through association with altered cell membrane glycoproteins. As patients with Borrmann type IV carcinoma are more likely to have lymph node metastases and peritoneal dissemination than those with other types (Maehara *et al*., 1992), STn may be an additional predictor of survival time for those patients.

Carbohydrate antigens with changes in glycosylation, detected in patients' serum, have been used as tumour

**Figure 2** The survival curves of patients with Borrmann type 4 gastric carcinoma. The thin black line indicates cases with STn-negative tumour and the bold black line indicates those with STn-positive tumour. **a**, Survival curves for all patients. There was a significant difference ($P<0.05$). **b**, Survival curves of 'curative' cases.

**Table IV** STn staining and pattern of recurrence

| Pattern of recurrence | STn staining Negative (n = 65) | Positive (n = 18) |
|---|---|---|
| Peritoneal dissemination | 23 | 5 |
| Metastasis to lymph nodes | 4 | 5 |
| Liver metastasis | 4 | 0 |
| Pulmonary metastasis | 2 | 0 |
| Bony metastasis | 0 | 1 |
| Local recurrence | 2 | 2 |
| Other recurrences | 3 | 0 |
| Total recurrences | 38 | 13 |
| No recurrence | 27 | 5 |

**Table V** STn staining and proliferative activity

| Proliferative activity | STn staining (mean ± s.d.) Negative (n = 123) | Positive (n = 57) |
|---|---|---|
| PCNA labelling (%) | 34.2 ± 13.2 | 41.5 ± 13.0** |
| AgNOR count | 3.48 ± 0.85 | 3.78 ± 0.98* |

*$P<0.05$, **$P<0.01$.

markers. As for STn antigen, it is not well known whether the levels of circulating serum STn antigen reflect changes in expression of this antigen on cancer cell membranes. The positive rates of STn antigen in immunohistochemical and serological study have been noted for some human cancers. In patients with colorectal cancer, the positive rate in tissue was 88% (112/128) (Itzkowitz *et al.*, 1990) and that in serum was 28% (5/18) (Motoo *et al.*, 1991). Those rates in patients with endometrial cancers were 84% (36/43) in tissue (Inoue *et al.*, 1991) and 5% (2/42) in serum (Inoue *et al.*, 1990). We found little documentation of comparisons of the STn levels in tissues and those in sera from the same patients. As for data on gastric cancer, together with data from our previous report (Takahashi *et al.*, 1993), we noted a relationship between the expression of STn by immunohistochemical staining and the serum STn antigen level. In about one-third of the cases of gastric cancer, cell-surface STn glycoconjugate seems to be drained into the systemic circulation. No particular characteristic emerged from cases of ready shedding of STn antigen into vessels. Patients with elevated serum STn antigen levels survived for only a short time, perhaps because of the higher tumour burden.

Even among patients with the same Borrmann type IV carcinoma, there are variations in lifespan. STn is associated with lymph node metastasis and peritoneal dissemination in this lesion, therefore this antigen may be one predictor of survival time for patients with Borrmann type IV carcinoma.

**Acknowledgements**

We thank M Ohara for helpful comments. This work was supported in part by a grant-in-aid from Kaibara Morikazu Medical Science Promotion Foundation, Japan.

**References**

ALTEVOGT P, FOGEL M, CHEINGSONG-POPOV R, DENNIS J, ROBINSON P AND SCHIRRMACHER V. (1983). Different patterns of lectin binding and cell surface sialylation detected on related high-and low-metastatic tumour lines. *Cancer Res.*, **43**, 5138-5144.

BORCHARD F. (1990). Classification of gastric carcinoma. *Hepatogastroenterology*, **37**, 223-232.

BORRMANN R. (1926). Geschwulste des Magens und des Duodenums. In *Handbuch Spez Pathol Anat und Histol IV/1*. Henke F and Lubarsch O (eds) pp. 812-1054. Springer: Berlin.

DIXON WJ. (1988). *BMDP Statistical Software Manual*, pp. 133-744. University of California Press: Berkeley, CA.

EGAN MJ AND CROCKER J. (1992). Nucleolar organiser regions in pathology. *Br. J. Cancer*, **65**, 1-7.

FURUKAWA H, HIRATSUKA M AND IWANAGA T. (1988). A rational technique for surgical operation on Borrmann type 4 gastric carcinoma: left upper abdominal evisceration plus Appleby's method. *Br. J. Surg.*, **75**, 116-119.

HAKOMORI S AND KANNAGI R. (1983). Glycosphingolipids as tumour-associated and differentiation markers. *J. Natl Cancer Inst.*, **71**, 231-251.

IMAMURA H, MORI T, OHKURA H, ISHII M, ARIYOSHI H, ENDO J, KITAO M, TAKEDA Y, KOBAYASHI H, INOUE M, HIROTA M, YAMAKIDO M, HAKOMORI S AND KANNAEI R. (1989). Basic and clinical evaluation of an immunoradiometric competitive inhibition assay for sialyl Tn antigen (in Japanese with English abstract). *Jpn J. Cancer Chemother.*, **16**, 3213-3219.

INOUE M, OGAWA H, NAKANISHI K, TANIZAWA O, KARINO K AND ENDO J. (1990). Clinical value of sialyl Tn antigen in patients with gynecologic tumours. *Obstet. Gynecol.*, **75**, 1032-1036.

INOUE M, OGAWA H, TANIZAWA O, KOBAYASHI Y, TSUJIMOTO M AND TSUJIMURA T. (1991). Immunodetection of sialyl-Tn antigen in normal, hyperplastic and cancerous tissues of the uterine endometrium. *Virchows Archiv. A., Pathol. Anat.*, **418**, 157-162.

ITZKOWITZ SH, BLOOM EJ, KOKAL WA, MODIN G, HAKOMORI S AND KIM YS. (1990). Sialosyl-Tn. A novel mucin antigen associated with prognosis in colorectal cancer patients. *Cancer*, **66**, 1960-1966.

KAKEJI Y, KORENAGA D, TSUJITANI S, HARAGUCHI M, MAEHARA Y AND SUGIMACHI K. (1991). Predicitive value of Ki-67 and argyrophilic nucleolar organizer region staining for lymph node metastasis in gastric cancer. *Cancer Res.*, **51**, 3503-3506.

KAKEJI Y, MAEHARA Y, ADACHI Y, BABA H, MOR M, FURUSAWA M AND SUGIMACHI K. (1994). Proliferative activity as a prognostic factor in Borrmann type 4 gastric carcinoma. *Br. J. Cancer*, **69**, 749-753.

KJELDSEN T, CLAUSEN H, HIROHASHI S, OGAWA T, IIJIMA H AND HAKOMORI S. (1988). Preparation and characterization of monoclonal antibodies directed to the tumour-associated O-linked sialosyl-2-6-N-acetylgalactosaminyl (sialosyl-Tn) epitope. *Cancer Res.*, **48**, 2214-2220.

KOBAYASHI H, TERAO T AND KAWASHIMA Y. (1992). Serum sialyl Tn as an independent predictor of poor prognosis in patients with epithelial ovarian cancer. *J. Clin. Oncol.*, **10**, 95-101.

MA XC, TERATA N, KODAMA M, JANCIC S, HOSOKAWA Y AND HATTORI T. (1993). Expression of sialyl-Tn antigen is correlated with survival time of patients with gastric carcinomas. *Eur. J. Cancer*, **29A**, 1820-1823.

McCORMICK D AND HALL PA. (1992). The complexities of proliferating cell nuclear antigen. *Histopathology*, **21**, 591-594.

MAEHARA Y, MORIGUCHI S, ORITA H, KAKEJI Y, HARAGUCHI M, KORENAGA D AND SUGIMACHI K. (1992). Lower survival rate for patients with gastric carcinoma of Borrmann type IV following gastric resection. *Surg. Gynecol. Obstet.*, **175**, 13-16.

MOTOO Y, KAWAKAMI H, WATANABE H, SATOMURA Y, OHTA H, OKAI T, MAKINO H, TOYA D AND SAWABU N. (1991). Serum sialyl-Tn antigen levels in patients with digestive cancers. *Oncology*, **48**, 321-326.

PASSANITI A AND HART GW. (1988). Cell surface sialylation and tumour metastasis. *J. Biol. Chem.*, **263**, 7591-7603.

PLOTON D, BOBICHON H AND ADNET JJ. (1982). Ultrastructural localization of NOR in nucleoli of human breast cancer tissues using a one-step Ag-NOR staining method. *Biol. Cell.*, **43**, 229-232.

SPRINGER GF. (1984). T and Tn, general carcinoma autoantigens. *Science*, **224**, 1198-1206.

TABUCHI Y, DEGUCHI H, IMANISHI K AND SAITOH Y. (1990). Immunohistochemical studies on the main entrance-route of CA19-9 into the peripheral venous blood of gastric cancer patients. *Cancer*, **66**, 1529-1533.

TAKAHASHI I, MAEHARA Y, KUSUMOTO T, YOSHIDA M, KAKEJI Y, KUSUMOTO H, FURUSAWA M AND SUGIMACHI K. (1993). Predictive value of preoperative serum sialyl Tn antigen levels in prognosis of patients with gastric cancer. *Cancer*, **72**, 1836-1840.

TAKAHASHI I, MAEHARA Y, KUSUMOTO T, KOHNOE S, KAKEJI Y, BABA H AND SUGIMACHI K. (1994). Combined evaluation of preoperative serum sialyl-Tn antigen and carcinoembryonic antigen levels is prognostic for gastric cancer patients. *Br. J. Cancer*, **69**, 163-166.

UICC (1987). *TNM Classification of Malignant Tumours*, 4th fully revised edition. Hermanek P and Sobin LH (eds) pp. 43-46. Springer: Berlin.

WERTHER JL, MACMURRAY SR, BRUCKNER H, TATEMATSU M AND ITZKOWITZ SH. (1994). Mucin-associated sialosyl-Tn antigen expression in gastric cancer correlates with an adverse outcome. *Br. J. Cancer*, **69**, 613-616.

YOGEESWARAN G AND SALK PL. (1981). Metastatic potential is positively correlated with cell surface sialylation of cultured murine tumor cell lines. *Science*, **212**, 1514-1516.


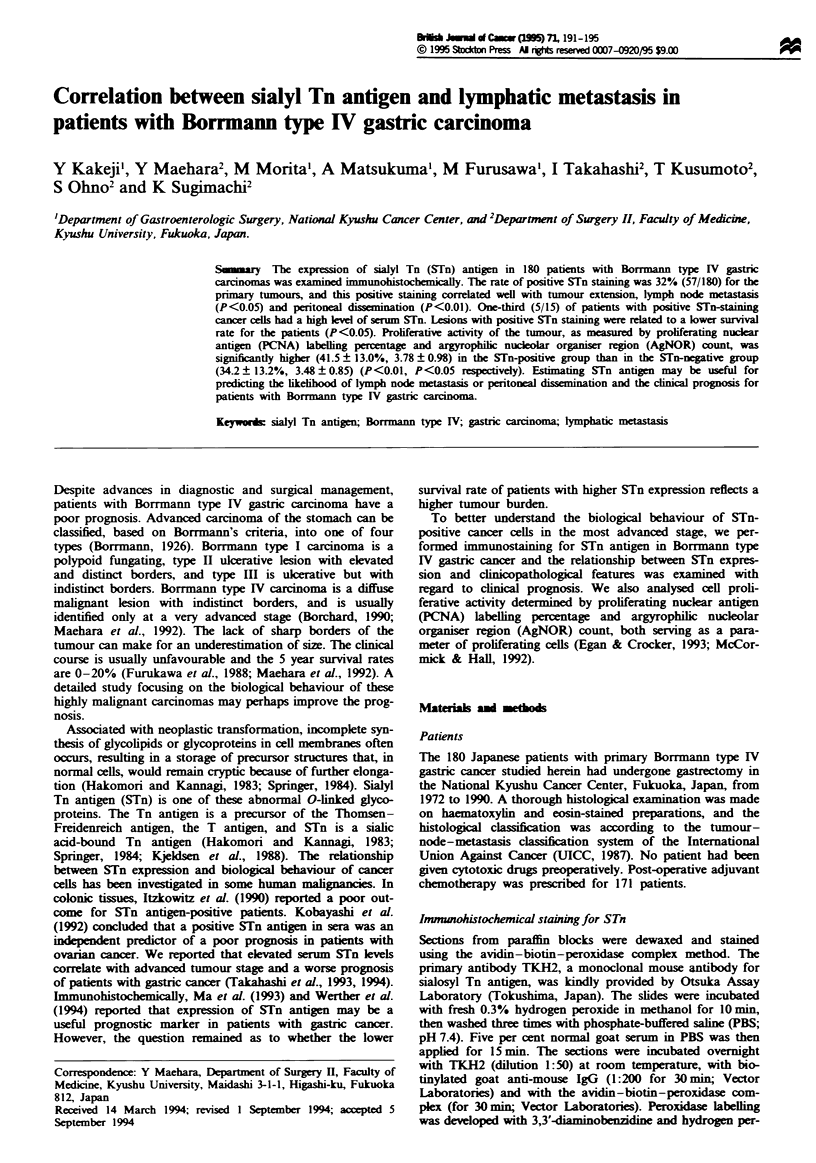

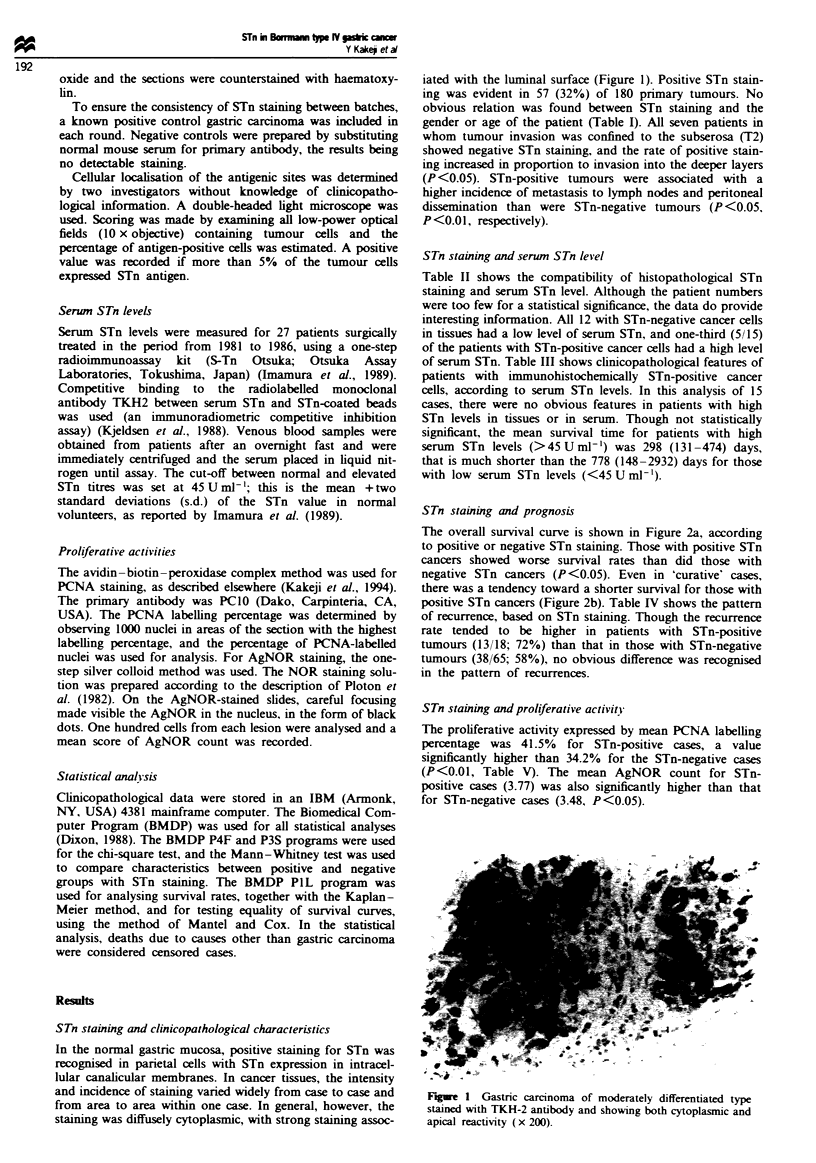

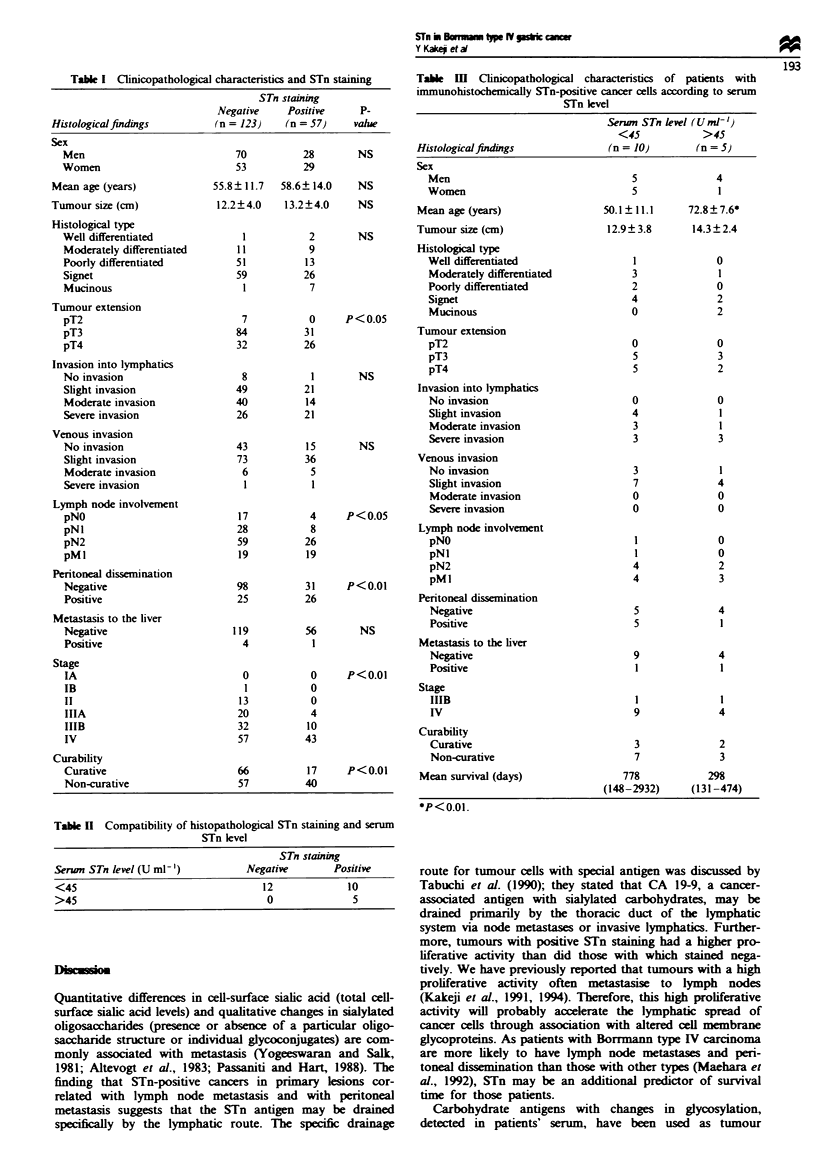

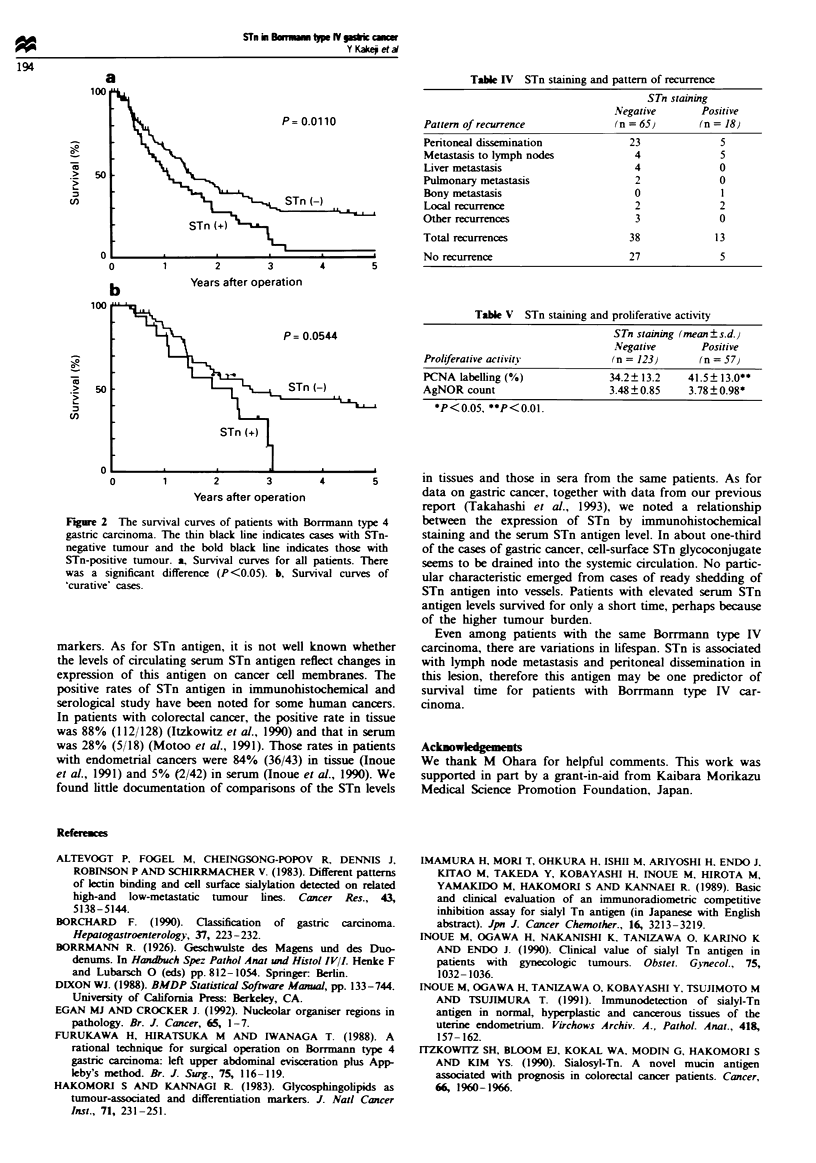

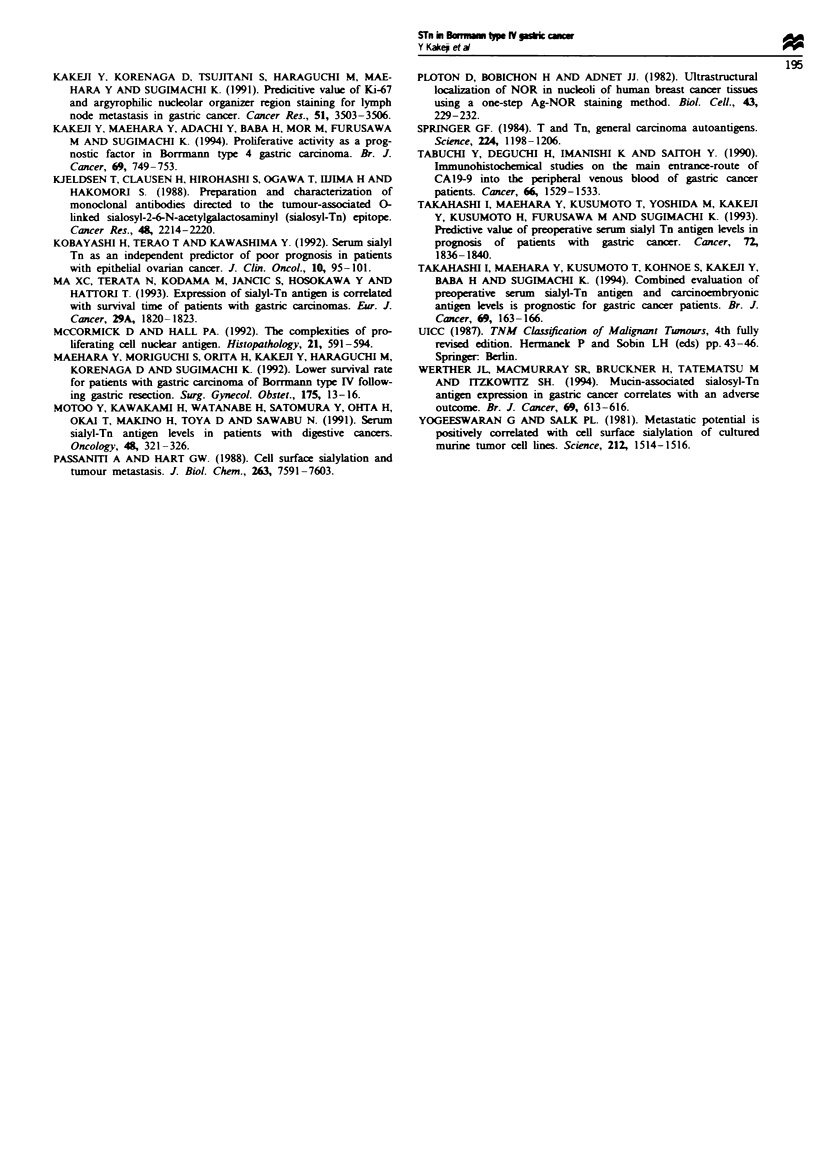


## References

[OCR_00770] Altevogt P., Fogel M., Cheingsong-Popov R., Dennis J., Robinson P., Schirrmacher V. (1983). Different patterns of lectin binding and cell surface sialylation detected on related high- and low-metastatic tumor lines.. Cancer Res.

[OCR_00779] Borchard F. (1990). Classification of gastric carcinoma.. Hepatogastroenterology.

[OCR_00796] Furukawa H., Hiratsuka M., Iwanaga T. (1988). A rational technique for surgical operation on Borrmann type 4 gastric carcinoma: left upper abdominal evisceration plus Appleby's method.. Br J Surg.

[OCR_00802] Hakomori S., Kannagi R. (1983). Glycosphingolipids as tumor-associated and differentiation markers.. J Natl Cancer Inst.

[OCR_00805] Imura H., Mori T., Ohkura H., Ishii M., Ariyoshi H., Endo J., Kitao M., Takeda I., Kobayashi H., Inoue M. (1989). [Basic and clinical evaluation of an immunoradiometric competitive inhibition assay for sialyl Tn antigen: (1). Evaluation of assay conditions and normal values. STN Study Group].. Gan To Kagaku Ryoho.

[OCR_00816] Inoue M., Ogawa H., Nakanishi K., Tanizawa O., Karino K., Endo J. (1990). Clinical value of sialyl Tn antigen in patients with gynecologic tumors.. Obstet Gynecol.

[OCR_00822] Inoue M., Ogawa H., Tanizawa O., Kobayashi Y., Tsujimoto M., Tsujimura T. (1991). Immunodetection of sialyl-Tn antigen in normal, hyperplastic and cancerous tissues of the uterine endometrium.. Virchows Arch A Pathol Anat Histopathol.

[OCR_00829] Itzkowitz S. H., Bloom E. J., Kokal W. A., Modin G., Hakomori S., Kim Y. S. (1990). Sialosyl-Tn. A novel mucin antigen associated with prognosis in colorectal cancer patients.. Cancer.

[OCR_00840] Kakeji Y., Korenaga D., Tsujitani S., Haraguchi M., Maehara Y., Sugimachi K. (1991). Predictive value of Ki-67 and argyrophilic nucleolar organizer region staining for lymph node metastasis in gastric cancer.. Cancer Res.

[OCR_00845] Kakeji Y., Maehara Y., Adachi Y., Baba H., Mori M., Furusawa M., Sugimachi K. (1994). Proliferative activity as a prognostic factor in Borrmann type 4 gastric carcinoma.. Br J Cancer.

[OCR_00851] Kjeldsen T., Clausen H., Hirohashi S., Ogawa T., Iijima H., Hakomori S. (1988). Preparation and characterization of monoclonal antibodies directed to the tumor-associated O-linked sialosyl-2----6 alpha-N-acetylgalactosaminyl (sialosyl-Tn) epitope.. Cancer Res.

[OCR_00858] Kobayashi H., Terao T., Kawashima Y. (1992). Serum sialyl Tn as an independent predictor of poor prognosis in patients with epithelial ovarian cancer.. J Clin Oncol.

[OCR_00861] Ma X. C., Terata N., Kodama M., Jancic S., Hosokawa Y., Hattori T. (1993). Expression of sialyl-Tn antigen is correlated with survival time of patients with gastric carcinomas.. Eur J Cancer.

[OCR_00871] Maehara Y., Moriguchi S., Orita H., Kakeji Y., Haraguchi M., Korenaga D., Sugimachi K. (1992). Lower survival rate for patients with carcinoma of the stomach of Borrmann type IV after gastric resection.. Surg Gynecol Obstet.

[OCR_00867] McCormick D., Hall P. A. (1992). The complexities of proliferating cell nuclear antigen.. Histopathology.

[OCR_00877] Motoo Y., Kawakami H., Watanabe H., Satomura Y., Ohta H., Okai T., Makino H., Toya D., Sawabu N. (1991). Serum sialyl-Tn antigen levels in patients with digestive cancers.. Oncology.

[OCR_00883] Passaniti A., Hart G. W. (1988). Cell surface sialylation and tumor metastasis. Metastatic potential of B16 melanoma variants correlates with their relative numbers of specific penultimate oligosaccharide structures.. J Biol Chem.

[OCR_00895] Springer G. F. (1984). T and Tn, general carcinoma autoantigens.. Science.

[OCR_00899] Tabuchi Y., Deguchi H., Imanishi K., Saitoh Y. (1990). Immunohistochemical studies on the main entrance-route of CA19-9 into the peripheral venous blood of gastric cancer patients. Correlation with CA19-9 levels in peripheral and portal blood.. Cancer.

[OCR_00912] Takahashi I., Maehara Y., Kusumoto T., Kohnoe S., Kakeji Y., Baba H., Sugimachi K. (1994). Combined evaluation of preoperative serum sialyl-Tn antigen and carcinoembryonic antigen levels is prognostic for gastric cancer patients.. Br J Cancer.

[OCR_00903] Takahashi I., Maehara Y., Kusumoto T., Yoshida M., Kakeji Y., Kusumoto H., Furusawa M., Sugimachi K. (1993). Predictive value of preoperative serum sialyl Tn antigen levels in prognosis of patients with gastric cancer.. Cancer.

[OCR_00922] Werther J. L., Rivera-MacMurray S., Bruckner H., Tatematsu M., Itzkowitz S. H. (1994). Mucin-associated sialosyl-Tn antigen expression in gastric cancer correlates with an adverse outcome.. Br J Cancer.

[OCR_00928] Yogeeswaran G., Salk P. L. (1981). Metastatic potential is positively correlated with cell surface sialylation of cultured murine tumor cell lines.. Science.

